# Delineation of capillary dropout in the deep retinal capillary plexus using optical coherence tomography angiography in a patient with Purtscher’s retinopathy exhibiting normal fluorescein angiography findings: a case report

**DOI:** 10.1186/s12886-016-0298-x

**Published:** 2016-07-19

**Authors:** Motoharu Tokimitsu, Masako Murata, Yuichi Toriyama, Takao Hirano, Yasuhiro Iesato, Toshinori Murata

**Affiliations:** Department of Ophthalmology, Shinshu University School of Medicine, 3-1-1 Asahi, Matsumoto, Nagano 390-8621 Japan; Department of Ophthalmology, Matsumoto Medical Center, National Hospital Organization, Matsumoto, Nagano Japan

**Keywords:** Purtscher’s retinopathy, Optical coherence tomography angiography, Capillary dropout, Fluorescein angiography, Deep retinal capillary plexus, Paracentral scotoma, Case report

## Abstract

**Background:**

Fat embolism in the deep retinal capillary plexus is one of the reported mechanisms underlying central/paracentral scotoma in patients with Purtscher’s retinopathy. Here we report the clear delineation of capillary dropout in the deep capillary plexus using optical coherence tomography angiography (OCTA) in a chronic case of unexplained scotoma that developed after femoral fracture. The patient exhibited normal fluorescein angiography (FA) findings and a normal retinal appearance.

**Case presentation:**

A 42-year-old Japanese man with a history of bilateral, unexplained paracentral scotoma that developed after femoral fracture and pulmonary fat embolism due to a car accident 20 years ago was referred to our outpatient clinic. Initial ophthalmological examination revealed unremarkable retinal findings. Goldmann perimetry, FA, and full field electroretinography showed no pathological changes. Although fat embolism in the retinal vasculature was suspected, psychosomatic visual field defects could not be ruled out. We performed OCTA, which clearly delineated capillary dropout in the deep retinal capillary plexus. A final diagnosis of paracentral acute middle maculopathy secondary to Purtscher’s retinopathy was made on the basis of this finding.

**Conclusions:**

Our findings suggest that OCTA clearly and noninvasively delineates the deep retinal capillary plexus and the superficial capillary plexus. Because conventional FA provides limited depth resolution, capillary dropout restricted within the deep capillary plexus cannot be detected, particularly when the superficial capillary plexus is well preserved. Thus, OCTA can be a useful tool for the detection of capillary dropout in the deep retinal capillary plexus.

## Background

Patients with Purtscher’s retinopathy develop paracentral or central scotoma after trauma at sites away from the eye, such as the chest and long bones. Dilated fundus examination typically shows cotton wool spots or patches of superficial retinal whitening that can be identified as hyper-reflective lesions in the inner retinal layers using spectral domain (SD) optical coherence tomography (OCT; SD-OCT). Several hypotheses have been proposed for the pathogenesis of retinal vascular occlusion, which is a key feature of Purtscher’s retinopathy. These include complement-induced granulocyte aggregation [1], air embolism associated with crushing chest injuries [2], and fat embolism associated with bone fractures [3].

However, characteristic fundus findings disappear gradually over several weeks to months. An immediate ocular examination is sometimes difficult after severe trauma, making the diagnosis of Purtscher’s retinopathy difficult. To detect changes in the normally appearing retina and diagnose Purtscher’s retinopathy in such chronic cases, decreased amplitude on multifocal electroretinography (ERG) [4], thinning of the inner retinal layers on SD-OCT [5], and fluorescein angiography (FA) findings have been used. While FA shows leakage in the region of the white retinal patches, disc edema, and venous staining in acute cases, capillary dropout/nonperfusion may be the only sign in chronic cases of Purtscher’s retinopathy [2, 6].

Paracentral acute middle maculopathy is a newly described entity in patients with acute-onset paracentral scotoma, who typically exhibit paracentral, hyper-reflective, band-like lesions in the inner nuclear layer (INL) of the macula, which progress to corresponding areas of severe INL thinning. This disease can be idiopathic or secondary to various diseases such as local retinal vascular disorders, systemic diseases, and trauma with associated Purtscher’s retinopathy [7]. Recently, OCT angiography (OCTA) has been reported to be a useful tool for the detection of capillary dropout in the deep capillary plexus in patients with paracentral scotoma in the chronic stage of paracentral acute middle maculopathy, which cannot be detected by FA because of its limited depth resolution. OCTA provides a high-resolution image of the microvasculature of the retina at various levels in the retina and choroid such as superficial capillary plexus, deep capillary plexus, outer retina, and choriocapillaris in the para- and peri-foveal areas [8, 9]. Here, we report a case involving a 42-year-old Japanese man who developed unexplained paracentral scotoma after suffering a femoral fracture in a car accident 20 years ago. Although his FA findings were normal, OCTA showed capillary dropout in the deep retinal capillary plexus, which led to a definite diagnosis of Purtscher’s retinopathy.

## Case presentation

A 42-year-old Japanese man presented with a history of bilateral paracentral scotoma that developed after he fractured his femur in a car accident 20 years ago. His best-corrected visual acuity (BCVA) was 20/20 in both eyes. He had been treated for the fracture and pulmonary embolism after the accident. He became aware of the symptoms of bilateral paracentral scotoma as soon as he regained consciousness. However, psychosomatic disease was suspected because Goldmann perimetry, FA, and full field ERG, brain computed tomography (CT) and magnetic resonance imaging (MRI) showed no apparent abnormalities. Consequently, observation was the only treatment, but the patient regularly sought ocular examinations at local eye clinics. In the follow-up, as the advent of new diagnostic tools, Humphry Field Analyzer demonstrated mild decrease in parafoveal sensitivity, and optical coherence tomography showed thinning of the parafoveal retina which provided the basis for the patient’s complaint of paracentral scotoma. He was eventually referred to our hospital because his eye symptoms gradually worsened and he feared central vision loss.

**Fig. 1:**
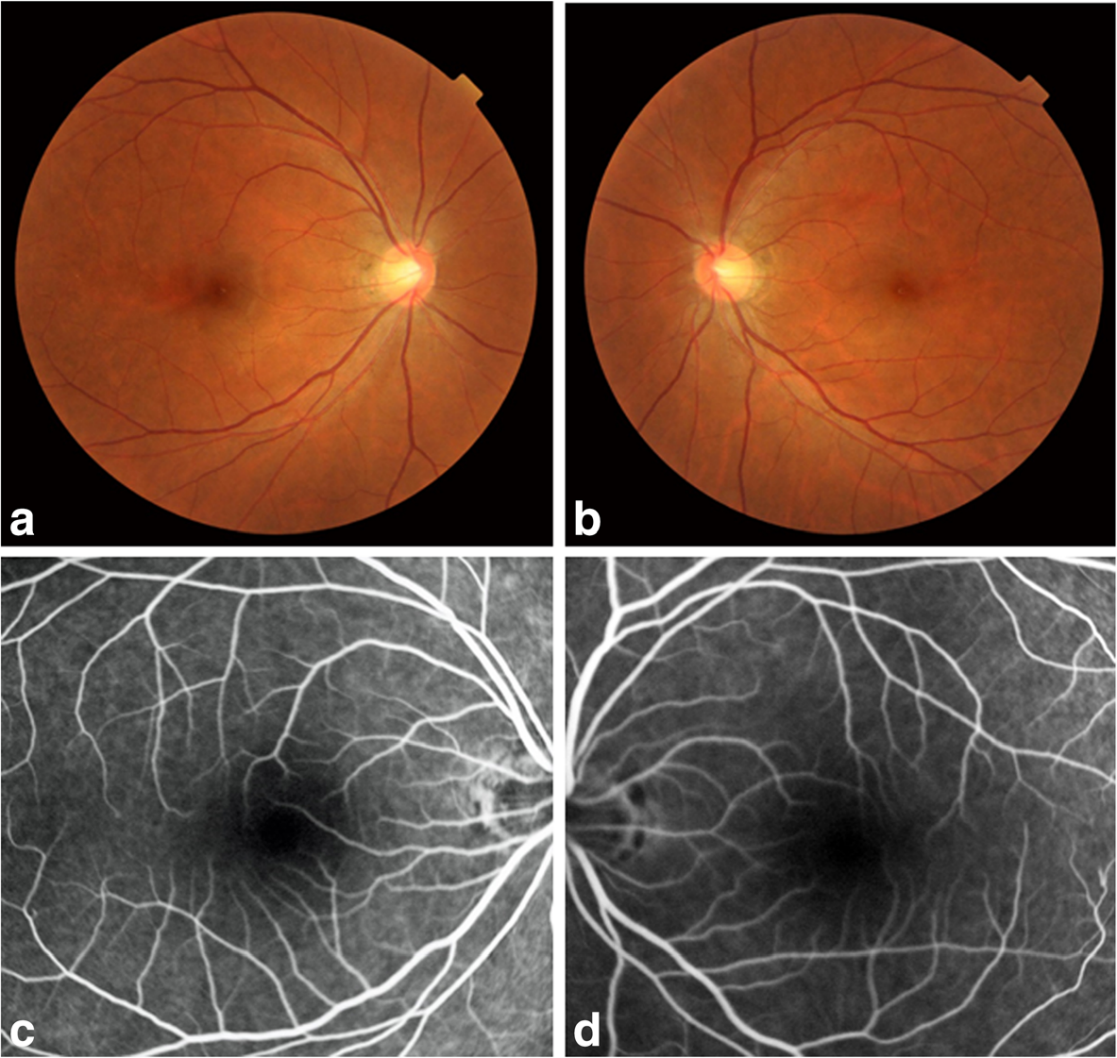
Fundus photographs and fluorescein angiograms of our 42-year-old Japanese patient with a 20-year history of bilateral paracentral scotoma secondary to trauma. The right (**a**) and left (**b**) fundi appear normal without hemorrhage or white patches. The fluorescein angiogram shows no leakage or no apparent capillary dropout in the right (**c**) and left (**d**) eyes

**Fig. 2:**
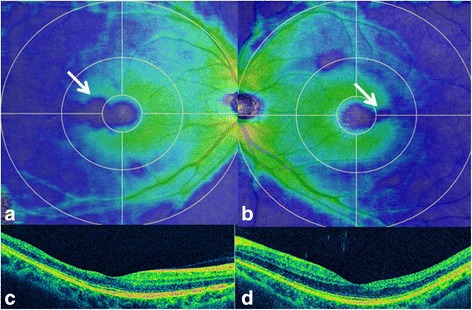
Optical coherence tomography findings for our 42-year-old Japanese patient with a 20-year history of bilateral paracentral scotoma secondary to trauma. The ganglion cell complex (GCC) shows thinning (blue; 250 μm) of the temporal macula (arrows) to the fovea (green; 300 μm) in the right (**a**) and left (**b**) eyes. Cross-sectional images also demonstrate temporal thinning of the inner retinal layers in the right (**c**) and left (**d**) eyes. The ellipsoid zone is well preserved in both eyes

**Fig. 3:**
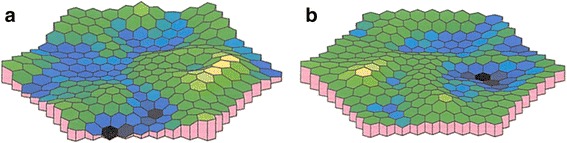
Multifocal electroretinography findings for our 42-year-old Japanese patient with a 20-year history of bilateral paracentral scotoma secondary to trauma. Decreased amplitude for the paracentral region is prominent in the right eye (**a**) and mild in the left eye (**b**)

On initial examination at our outpatient clinic, anterior segment and dilated fundus findings were unremarkable, with no retinal hemorrhage or cotton wool spots (Fig. 1a, b). Because of his history of pulmonary fat embolism after the car accident, chronic embolism in the retinal circulation was suspected. However, FA performed using the SPECTRALIS® HRA device (Heidelberg Engineering, Heidelberg, Germany) showed no leakage or capillary dropout (Fig. 1c, d). Goldmann perimetry failed to detect paracentral scotoma, whereas the Humphrey field analyzer (Carl Zeiss, San Leandro, California, USA) demonstrated a mild decrease in retinal sensitivity in the paracentral regions. However, a gray scale demonstrated a subnormal pattern in both eyes. Both the ganglion cell complex (GCC; Fig. 2a, b) assessed on macular scans (RS-3000, NIDEK, Gamagori, Japan) and cross-sectional SD-OCT (CIRRUS 5000, Carl Zeiss, San Leandro, California, USA) images (Fig. 2c, d) showed marked thinning of the inner retinal layers in the temporal macular region. Multifocal ERG also showed a decreased amplitude for the corresponding regions (Fig. 3a, b).

**Fig. 4:**
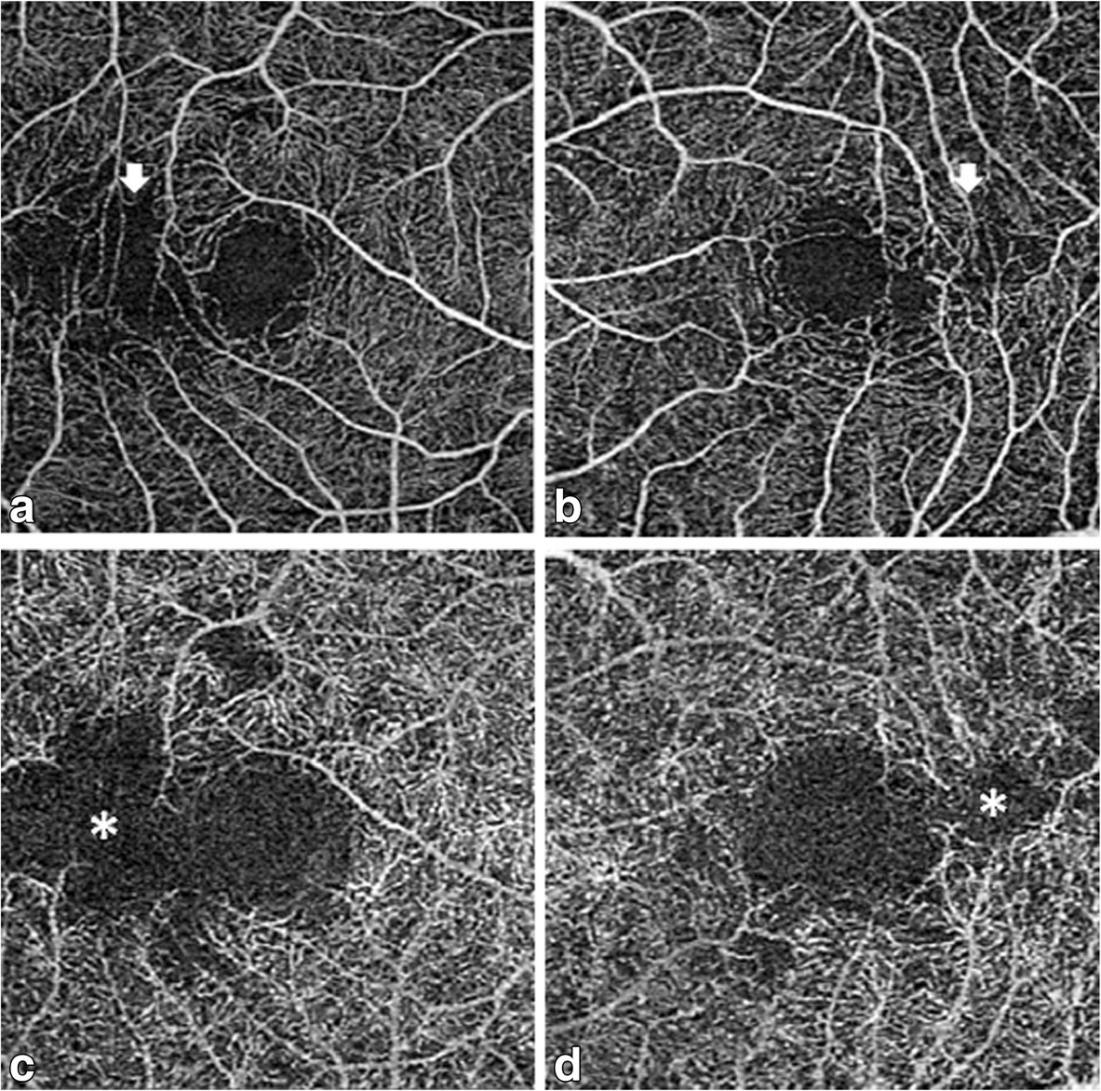
Optical coherence tomography angiography findings for our 42-year-old Japanese patient with a 20-year history of bilateral paracentral scotoma secondary to trauma. The superficial retinal capillary plexus in the right (**a**) and left (**b**) eyes shows minimal capillary dropout in the temporal region to the foveal avascular zone (arrows). The deep capillary plexus shows marked capillary dropout in the corresponding region in the right (**c**) and left (**d**) eyes (asterisks)

We then performed OCTA (AngioPlex CIRRUS 5000, Carl Zeiss, San Leandro, California, USA), which clearly demonstrated capillary dropout in the regions corresponding to inner retinal layer thinning. Capillary loss was mild in the superficial capillary plexus (Fig. 4a, b) and more prominent in the deep retinal capillary plexus (Fig. 4c, d). On the basis of these findings, a final diagnosis of paracentral acute middle maculopathy secondary to Purtscher’s retinopathy was made.

## Conclusions

Fluorescein angiography has been the method of choice for imaging the retinal/choroidal vessels and diagnosing vascular diseases. OCTA is a novel device that visualizes the vasculature utilizing a contrast generated by differentiating between moving cells in the vessels and the static surrounding tissue. Consequently, injecting intravenous dye is not necessary, thus avoiding potential risks of nausea or other serious adverse events. Its clinical use is currently widely investigated [8, 9].

We reported the clear delineation of capillary dropout using OCTA in a patient with Purtscher’s retinopathy exhibiting normal FA findings. FA has been the gold standard for imaging capillary dropout/nonperfusion, although its depth resolution is not enough for the identification of isolated loss of the deep capillary plexus. Consequently, FA shows typically normal or subnormal findings for eyes with paracentral acute middle maculopathy, in whom focal occlusion of the deep capillary plexus attributes to the formation of band-like lesions in INL, as observed on cross-sectional SD-OCT images. Our patient became aware of the symptoms of paracentral scotoma soon after a car accident 20 years ago. However, Goldmann perimetry did not detect paracentral visual field defects and FA showed normal findings. Because all ophthalmological and systemic evaluations after the accident, including brain CT and MRI, showed normal findings, psychosomatic disease had been suspected. However, the patient persistently visited local eye clinics and underwent newly developed examinations as they were introduced. Pattern deviation observed using the Humphrey field analyzer indicated a mild decrease in retinal sensitivity in the regions corresponding to those affected by paracentral scotoma. SD-OCT showed focal thinning of the inner retinal layers in the corresponding regions, while multifocal ERG showed a decrease in amplitude. Although we suspected Purtscher’s retinopathy due to fat embolism as the pathogenesis of paracentral scotoma, FA failed to provide direct evidence of capillary dropout. Finally, OCTA detected capillary dropout in the outer capillary plexus in the region responsible for the visual field defect. An important feature of OCTA is immediate visualization of retinal capillary dropout, while thinning of the inner retinal layers and amplitude reduction were only indicative of vascular changes secondary to fat embolism.

In conclusion, the findings from our case suggest that OCTA is a useful tool for the visualization of small capillary dropout in the deep retinal capillary plexus, which cannot be demonstrated by FA because of limited depth resolution, particularly when the superficial capillary plexus is well preserved. OCTA clearly and noninvasively delineates the deep retinal capillary plexus and the superficial capillary plexus separately, which aids in diagnosis of Purtscher’s retinopathy. Further investigation with increased number of patients is warranted.

## Abbreviations

BCVA, best-corrected visual acuity; CT, computed tomography; ERG, electroretinography; FA, fluorescein angiography; GCC, ganglion cell complex; INL, inner nuclear layer; MRI, magnetic resonance imaging; OCT, optical coherence tomography; OCTA, optical coherence tomography angiography; SD-OCT, spectral domain optical coherence tomography
